# Antimicrobial susceptibilities of specific syndromes created with organ-specific weighted incidence antibiograms (OSWIA) in patients with intra-abdominal infections

**DOI:** 10.1186/s12879-018-3494-x

**Published:** 2018-11-19

**Authors:** Lianxin Liu, Yuxing Ni

**Affiliations:** 1 Department of Hepatobiliary Surgery, the First Affiliated Hospital of Harbin Medical University. Key Laboratory of Hepatosplenic Surgery, Ministry of Education, No. 23 Youzheng Street, Harbin, 150001 China; 2 Department of Hospital Infection Control, Rui Jin Hospital, Shanghai Jiao Tong University School of Medicine, No. 197 Rui-Jin 2nd Road, Shanghai, 200025 China

**Keywords:** SMART, Intra-abdominal infection, Gram-negative bacteria, Antibiotics, Organ-specific weighted incidence antibiogram (OSWIA), Organ-specific susceptibility

## Abstract

**Background:**

The aim was to evaluate the value of organ-specific weighted incidence antibiogram (OSWIA) percentages for bacterial susceptibilities of Gram-negative bacteria (GNB) collected from intra-abdominal infections (IAIs) during SMART 2010–2014.

**Methods:**

We retrospectively calculated the OSWIA percentages that would have been adequately covered by 12 common antimicrobials based on the bacterial compositions found in the appendix, peritoneum, colon, liver, gall bladder and pancreas.

**Results:**

The ESBL positive rates were 65.7% for *Escherichia coli*, 36.2% for *Klebsiella pneumoniae*, 42.9% for *Proteus mirabilis* and 33.1% for *Klebsiella oxytoca. Escherichia coli* were mainly found in the appendix (76.8%), but less so in the liver (32.4%). *Klebsiella pneumoniae* constituted 45.2% of the total liver pathogenic bacteria and 15.2–20.8% were found in 4 other organs, except the colon and appendix (< 10%). The percentages of *Pseudomonas aeruginosa* infections were higher in the gall bladder, intra-abdominal abscesses, pancreas and colon (10.2–13.2%) and least (5.4%) in the appendix. The susceptibilities of hospital acquired (HA) and community acquired (CA) IAI isolates from appendix, gall bladder and liver showed ≥80% susceptibilities to amikacin (AMK), imipenem (IPM), piperacillin-tazobactam (TZP) and ertapenem (ETP), while the susceptibility of isolates in abscesses and peritoneal fluid showed ≥80% susceptibility only to amikacin (AMK) and imipenem (IPM). In colon CA IAI isolates susceptibilities did not reach 80% for AMK and ETP, and in pancreatic IAIs susceptibilities of HA GNBs did not reach 80% to AMK, TZP and ETP, and CA GNBs to IMP and ETP. In addition, besides circa 80% susceptibility of HA and CA IAI isolates from appendix to cefoxitin (FOX), IAI isolates from all other organs had susceptibilities between 7.6 and 67.9% to all cephalosporins tested, 28.3–75.2% to fluoroquinolones and 7.6–51.0% to ampicillin-sulbactam (SAM), whether they were obtained from CA or HA infections.

**Conclusion:**

The calculated OSWIA susceptibilities were specific for different organs in abdominal infections.

**Electronic supplementary material:**

The online version of this article (10.1186/s12879-018-3494-x) contains supplementary material, which is available to authorized users.

## Background

The massive over prescribing of antimicrobial agents has led to dramatic changes in clinical susceptibilities to antibiotics and multidrug-resistant (MDR) infection has been proven to be one of the major causes of mortality, especially those patients with IAIs [1, 2]. Mortality rates associated with secondary peritonitis and severe sepsis or septic shock average approximately 30%. [3, 4]

In 2012, Tabah et al. conducted a prospective, multicentre non-representative cohort study in 162 intensive care units (ICUs) in 24 countries, and showed that MDR and pan-drug-resistance (PDR) was increased in Europe, and Gram-negative bacterial infections were especially associated with an increased 28-day mortality [5]. Lack of effective initial empirical antimicrobial treatment within 24 h increases mortality significantly compared with appropriate antimicrobial treatment (63.0–65.2% vs 30.6–42.0%) [6, 7]. It has also been noted that effective empirical treatment needs to be supported by epidemiological studies and antimicrobial susceptibility data about the prevalence of local pathogenic bacteria. However, traditional epidemiological studies are limited to the description of the broad bacterial distributions and variable drug susceptibilities in individual hospitals, while detailed organ-specific data are usually lacking. Recently, Herbert et al. (2012) developed a novel method of displaying microbiology data to support early empirical antimicrobial treatments, which they termed the weighted-incidence syndromic combination antibiogram (WISCA). It classifies patients by syndrome and determines, for each patient with a given syndrome, whether a particular treatment regimen (one or more drugs) would have covered all the organisms recovered from their infections [8]. These data are calculated by dividing the number of the patients treated with a particular antimicrobial drug by the total number of patients. In the present study we created OSWIAs, which estimated the probability of organ specific isolates being susceptible to particular antibiotics.

Using data from the SMART study, we analyzed organ specific antimicrobial susceptibilities of Gram-negative bacteria in abdominal infections via OSWIA determinations in order to explore the practicability of this protocol and to assess its potential benefits in clinical practice in China.

## Materials and methods

The Human Research Ethics Committee of Peking Union Medical College Hospital approved this study and waived the need for consent (Ethics Approval Number: SK238). Patient data were collected from a total of 21 hospitals in 16 Chinese cities from 2010 to 2014 and according to the SMART protocol each participating hospital provided at least 100 consecutive aerobic and facultative Gram-negative bacilli from patients with IAIs excluding duplicate isolates.

Isolates (8066) of Gram-negative aerobic bacteria and other pathogenic bacteria were obtained from different infected abdominal organs, including fermentative and non-fermentative bacteria in the appendix, peritoneum, colon, liver, gall bladder and pancreas from 2012 to 2014. All duplicate isolates (the same genus and species from the same patient) were excluded. Isolates collected within 48 h of hospitalization were categorized as community acquired (CA) IAIs, and those collected after 48 h were categorized as hospital acquired (HA) IAIs. The majority of intra-abdominal specimens were obtained during surgery, though some paracentesis specimens were also collected.

### Bacterial identification and antimicrobial susceptibility testing

Bacteria were identified by standard methods used in the participating clinical microbiology laboratories and all organisms were deemed clinically significant according to local criteria. All isolates were sent to the central clinical microbiology laboratory of Peking Union Medical College Hospital for re-identification using MALDI-TOF MS (Vitek MS, BioMérieux, France).

To assess antimicrobial susceptibilities, minimum inhibitory concentrations (MICs) were determined with dehydrated MicroScan broth micro dilution panels (Siemens Medical Solutions Diagnostics, West Sacramento, CA, USA), according to the guidelines of the 2012 Clinical and Laboratory Standards Institute (CLSI) [9]. Susceptibility interpretations were based on the CLSI M100-S23 clinical breakpoints [10], and the ATCC 25922 strain of *Escherichia coli* (*E. coli*), the ATCC 27853 strain of *Pseudomonas aeruginosa* (*P. aeruginosa)*, and the ATCC 700603 strain of *Klebsiella pneumoniae* (*K. pneumoniae*) were used as reference strains in each set of MIC tests for quality control. The antibiotics tested were the aminoglycoside amikacin (AMK), the carbapenems ertapenem (ETP) and imipenem (IPM), the cephamycins cefoxitin (FOX), ceftazidime (CAZ), cefepime (FEP), cefotaxime (CTX) and ceftriaxone (CRO), the fluoroquinolones levofloxacin (LVX) and ciprofloxacin (CIP) as well as the broad spectrum penicillins combined with β-lactamase inhibitors ampicillin-sulbactam (SAM) and piperacillin-tazobactam (TZP).

Phenotypic identification of extended-spectrum β-lactamase (ESBL) positive bacteria were carried out by CLSI recommended methods [10]. If MICs were ≥ 2 μg/mL for cefotaxime or ceftazidime, the MICs of cefotaxime or ceftazidime plus clavulanic acid (4 μg/mL) were determined and ESBL production was defined as a ≥ 8-fold decrease of MICs for cefotaxime or ceftazidime plus clavulanic acid.

### Organ-specific weighted incidence antibiogram (OSWIA) calculation

We evaluated the data retrospectively and analyzed the pathogenic bacteria distribution in various abdominal organs. OSWIAs were calculated using the following equation: Weighted susceptibility of a certain antimicrobial drug in a certain organ = antimicrobial susceptibility of A × the constituent ratio of A in the organ + antimicrobial susceptibility of B × the constituent ratio of B in the organ + antimicrobial susceptibility of C × the constituent ratio of C in the organ + (where A, B, C represent the pathogenic bacteria in a certain organ).

### Statistical analysis

The susceptibility of all Gram-negative isolates combined was calculated using breakpoints appropriate for each species and assuming 0% susceptibility for species with no breakpoints for any given drug. The 95% confidence intervals (CIs) were calculated using the adjusted Wald method; linear trends of ESBL rates in different years were assessed for statistical significance using the Cochran-Armitage test and comparison of ESBL rates were assessed using a chi-squared test. *P*-values < 0.05 were considered to be statistically significant.

## Results

### Distribution of gram-negative enteric bacteria from 2010 to 2014

The majority if IAI isolates included *E. coli*, with 3764 strains in total (46.7%), of which 2472 (65.7%) were ESBL-producing strains, followed by *K. pneumoniae* with 1486 strains in total (18.4%) of which 538 (36.2%) were ESBL-producing strains. Other major pathogenic bacteria included 804 strains of *P. aeruginosa* (10.0%) and 558 strains of *Acinetobacter baumannii* (*A. baumannii*) (6.9%), which both belong to the non-fermentative bacteria group, as well as 410 strains of *Enterobacter cloacae (E. cloacae)* (5.1%). The rest of the pathogenic bacteria comprised < 2% of the total. The majority of non-fermentative GNBs was isolated from HA IAIs (Table 1). A total of 61 other strains were rarely isolated and detailed information is listed in Additional file 1: Table S1.

**Table 1:** Distribution of pathogenic Gram-negative bacteria responsible for IAIs (2010–2014)

Organism	Sum (%)
*Fermentative bacteria*	
*Escherichia coli*	3764 (46.7%)
ESBL-producing strains (of % *E. coli*)	2.472 (65.7%)
*Klebsiella pneumoniae*	1.486 (18.4%)
ESBL-producing strains (of % *K. pneumoniae*)	538 (36.2%)
*Enterobacter cloacae*	410 (5.1%)
*Proteus mirabilis*	147 (1.8%)
ESBL-producing strains (of % *P. mirabilis*)	63 (42.9%)
*Enterobacter aerogenes*	138 (1.7%)
*Citrobacter freundii*	138 (1.7%)
*Klebsiella oxytoca*	124 (1.5%)
ESBL-producing strains (of % *K. oxytoca*)	41 (33.1%)
*Morganella morganii*	93 (1.2%)
*Serratia marcescens*	53 (0.7%)
*Aeromonas hydrophila*	35 (0.4%)
*Proteus vulgaris*	22 (0.3%)
*Citrobacter braakii*	21 (0.3%)
*Citrobacter koseri*	17 (0.2%)
*Non-Fermentative bacteria*	
*Pseudomonas aeruginosa*	804 (10.0%)
*HA*	636 (79.1)
*CA*	162 (20.1)
*Acinetobacter baumannii*	558 (6.9%)
*HA*	451 (80.8)
*CA*	101 (18.1)
*Stenotrophomonas maltophilia*	86 (1.1%)
*HA*	66 (76.7)
*CA*	20 (23.3)
Other^a^	170 (2.1%)
Total	8066

^a^Other includes < 0.2% of *Enterobacteriaceae* or < 1.1% of non-fermentative bacterial strains isolated from the IAIs (*n* = 61)

### Comparison of the pathogenic distribution of abdominal infections in different organs (2010–2014)

**Fig. 1:**
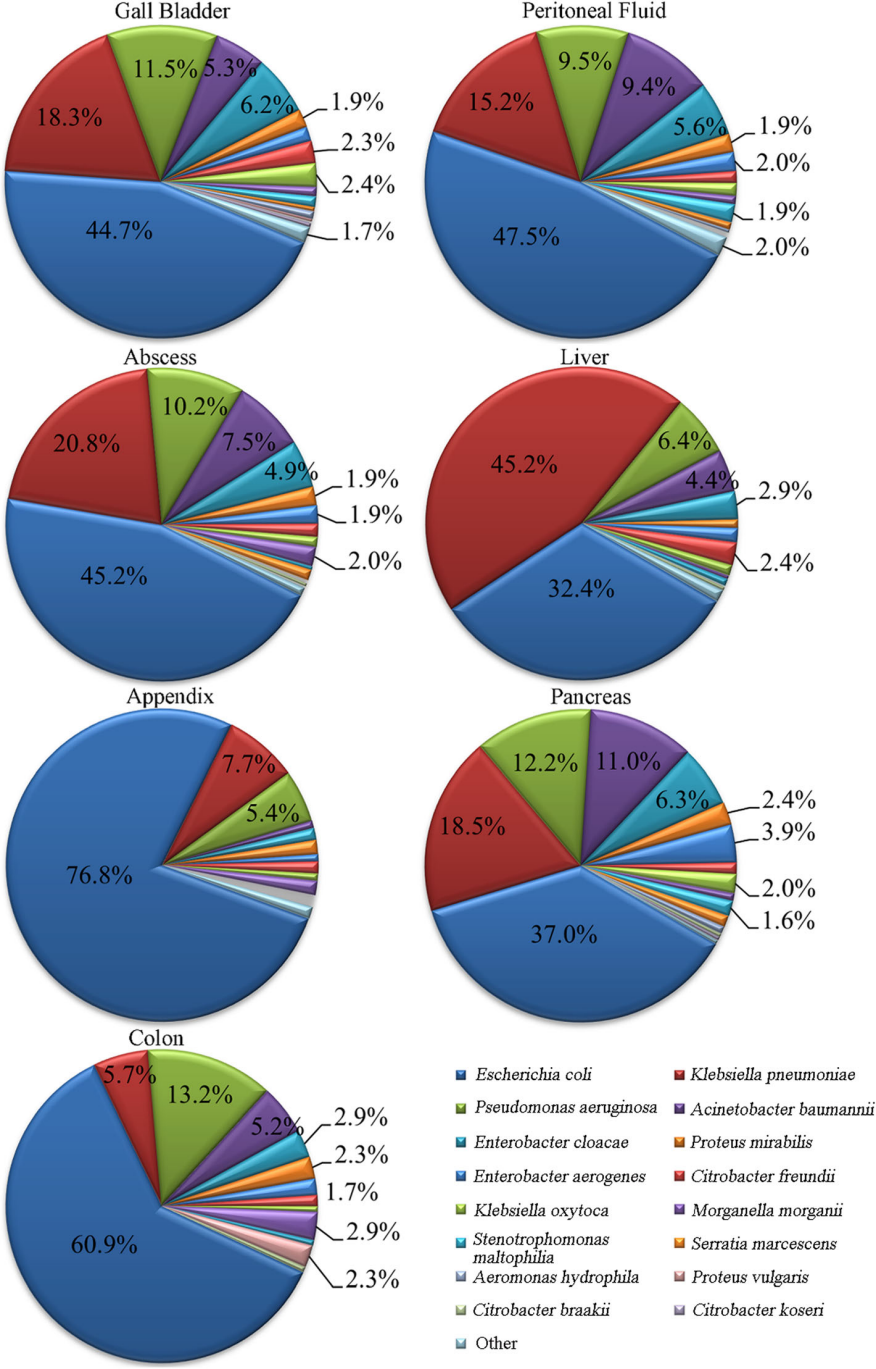
Composition of pathogenic bacteria in infected abdominal organs from 2010 to 2014

In Figure 1, we show the pathogenic distribution of Gram-negative bacteria in some infected organs in the abdomen, including 2510 strains from the gall bladder (31.1%), 2078 strains from peritoneal fluid (25.8%), 1444 strains from abdominal abscesses (17.9%), and the remainder from the appendix (405 strains), colon (174 strains), liver (553 strains) and pancreas (256 strains), respectively.

The majority of the abdominal pathogenic bacteria included fermentative bacteria comprising *E. coli*, *K. pneumoniae*, and the non-fermentative bacteria *A. baumannii* and *P. aeruginosa*. The highest percentage of *E. coli* was found in the appendix (76.8%) and the least percentage in the liver (32.4%). *K. pneumoniae* accounted for 45.2% of the total pathogenic bacteria in the liver and moderate fractions (15.2–20.8%) in the gall bladder, peritoneal fluid, abscesses and pancreas, but only 7.7% in the appendix and 5.7% in the colon. *P. aeruginosa* was one of the major pathogens found in the gall bladder (11.5%), abscesses (10.2%), pancreas (12.2%) and colon (13.2%). The highest percentage of *A. baumannii* was found in the pancreas (11.0%) and the least percentage in the appendix (≤ 1%) (Fig. 1).

Non-fermentative GNBs accounted for 12.5% of liver and 6.7% of appendix infections, whereas the percentages in other organs were 18.8–25.8%. More non-fermentative bacteria were found in HA compared to CA infections in all abdominal organs except the appendix. In pancreas infections, the ESBL producing rates of *Enterobacteriaceae* were slightly higher in HA compared to CA IAIs, but in the liver the GNB rate was almost double. There were obvious differences between *Enterobacteriaceae* ESBL producing rates within the organs, being highest in the colon followed by the pancreas and peritoneal fluid infections (Table 2).

**Table 2:** HA and CA IAI isolate distributions of *Enterobacteriaceae* and non-fermenting GNBs in the indicated organs

	Non-fermenting GNBs^a^ N (%)		*Enterobacteriaceae* N (%)				
	HA	CA	HA		CA		Total
				ESBL+ (% of HA)		ESBL+ (% of CA)	
Gall bladder^b^	390 (15.5)	84 (3.4)	1593 (63.5)	701 (44.0)	438 (17.5)	162 (37.0)	2510
Peritoneal fluid^c^	375 (18.1)	82 (4.0)	1184 (57.0)	635 (53.6)	415 (20.0)	196 (47.2)	2078
Abscess^d^	225 (15.6)	46 (3.2)	891 (61.7)	434 (48.7)	281 (19.5)	133 (47.3)	1444
Liver	59 (10.7)	10 (1.8)	378 (68.4)	153 (40.5)	106 (19.2)	23 (21.7)	553
Appendix	11 (2.7)	16 (4.0)	146 (36.1)	72 (49.3)	232 (57.3)	101 (43.5)	405
Pancreas	55 (21.5)	11 (4.3)	155 (60.6)	86 (55.5)	35 (13.7)	21 (60)	256
Colon	23 (13.2)	10 (5.8)	122 (70.1)	81 (66.4)	19 (10.9)	12 (63.2)	174

^a^There was no ESBL+ isolates in non-fermenting GNBs

^b^There were 5 unidentified isolates in the *Enterobacteriaceae*

^c^There were 9 unidentified isolates in non-fermenting GNB and 13 not identified isolates in the *Enterobacteriaceae*

^d^There was 1 unidentified isolate in the *Enterobacteriaceae*

*Antimicrobial susceptibilities of specific syndromes determined by OSWIAs in IAIs.*

**Fig. 2:**
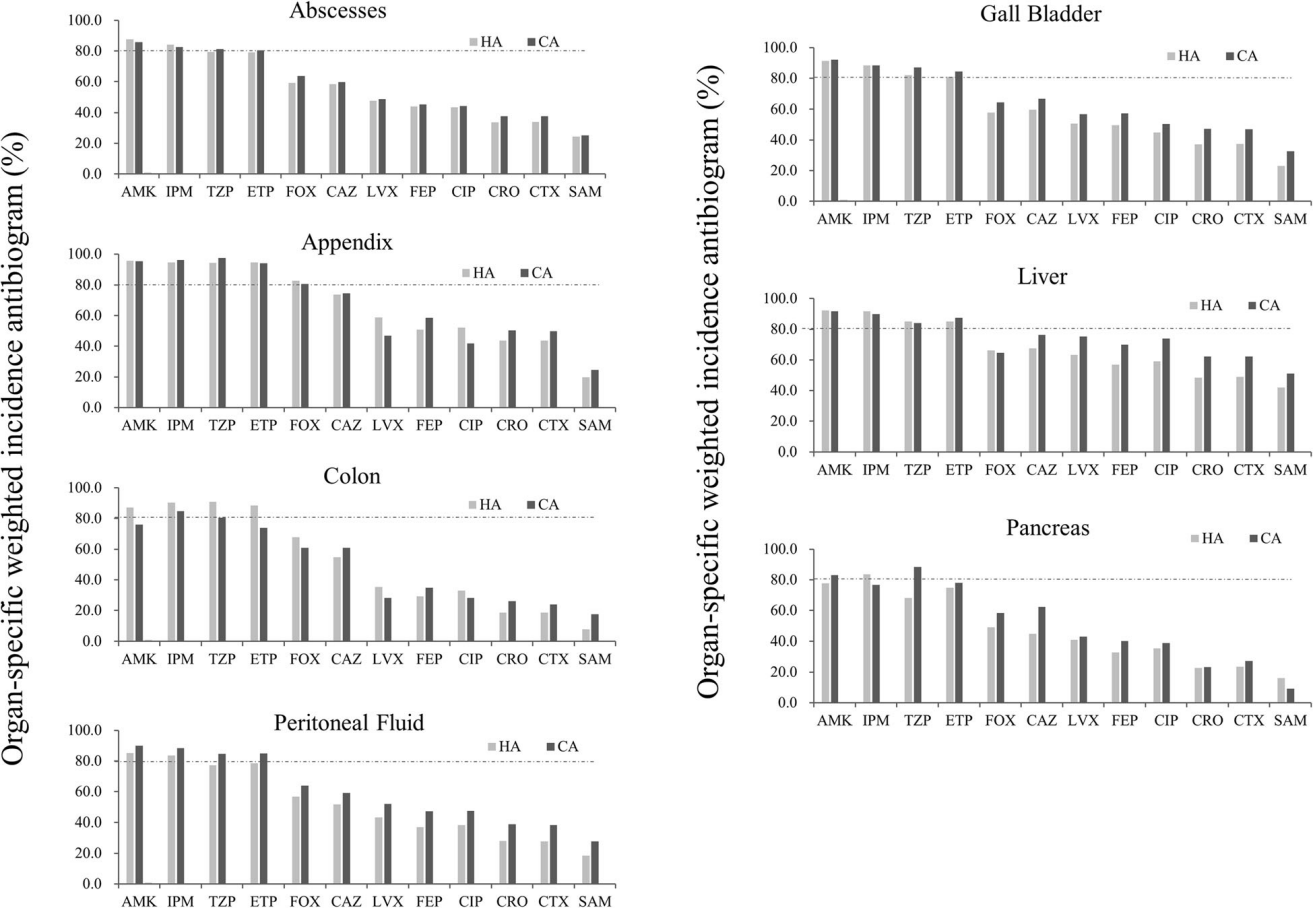
Comparison of the antimicrobial susceptibility rates calculated by the OSWIA method for all GNB infections in the indicated abdominal organs

Next, we calculated the OSWIAs (Fig. 2). Additionally, apart from the liver, all other organs presented with a typical “stair-step” shape, with only AMK, IPM, TZP and ETP susceptibilities being ≥80%; the rest of the antibiotics had activity far below this level. The highest susceptibility rates to AMK, IMP, TZP and ETP were found in he appendix and differences between HA and CA IAI susceptibilities were more pronounced in the colon, peritoneal fluid and pancreas, being higher in CA derived strains from peritoneal fluid and pancreas but less in CA strains isolated from colon infections. Apart from susceptibility of appendix isolates to FOX, IAI isolates from all other organs were susceptible (18–74.5% to all cephalosporins tested including cefoxitin, whether they were obtained from CA or HA infections, suggesting a high prevalence of ESBL production. Susceptibilities to fluoroquinolones were 28.3–75.2% and to SAM 7.6–51.0% (Fig. 2).

## Discussion

A timely worldwide multi-center cross-sectional study showed that abdominal infections constituted 19.6% of infected patients in ICUs. The mortality of patients was higher than those with other infections (29.4% vs 24.4%, *P* < 0.001). Nearly all patients were treated with antibiotics (98.1%), but the results of microbial culture were obtained in only about two-thirds of patients [11]. The common use of empirical antimicrobial drugs needs to refer to local epidemiological studies and antimicrobial susceptibility data [12]. Traditional epidemiological studies are conducted in a “bacteria-antibiotics” mode, which first describes the isolated local bacteria strains, then reports the corresponding drug susceptibilities. In 2012, Herbert et al. proposed that WISCAs could determine the likelihood that a specific regimen can effectively treat all organisms in a patient with a specific syndrome after microbial and clinical data analysis [8]. This method is based on significant differences in the distribution of pathogenic bacteria sites, which is usually lacking in traditional microbial/epidemiological studies; thus, the patients cannot be treated precisely.

In addition, OSIWA estimates for empirical therapies of IAIs might serve as an initial hint for the choice of antibiotics when other information is not available, since bacterial strain identification and antibiograms requires some time to produce the data, particularly for IAIs.

SMART is a global multi-center abdominal infection program that mainly monitors Gram-negative bacteria and their susceptibility to antibiotics. The data showed that *Enterobacteriaceae* were still the major strains found in abdominal infections (2010–2014) in China, the most common types being *E. coli* and *K. pneumoniae*, which is in agreement with a previous study [13].

Other non-fermentative bacteria include *P. aeruginosa* and *A. baumannii*, which accounted for 10.0 and 6.9% of pathogens, respectively. *A. baumannii* were not commonly detected in general abdominal infections, but its distribution rate was high in certain organs such as the pancreas. According to our analyses, the composition of pathogenic Gram-negative bacteria isolated from the abdominal cavity is different, based on the isolation sites. For example, Gram-negative *E. coli* bacteria from the appendix accounted for 76.8%, but was distributed < 40% in the liver and pancreas. *K. pneumoniae* accounted for 45.2% of the total pathogenic bacteria in the liver but < 6% in the colon, which is in line with previous reports that liver infections caused by *K. pneumoniae* are increasing [14, 15].

Therefore, the distribution of pathogenic bacteria in different abdominal organs should be considered in empirical therapy. We further analyzed comprehensive antimicrobial susceptibilities using the OSWIA algorithm, which is calculated according to the susceptibility of each bacterium to a specific drug times the sum of the total proportion of the bacterium present in a specific infection. We found that OSWIA closely matched the clinical data: compared with pancreatitis and other infection sites, appendicitis had a higher overall antimicrobial susceptibility. Additionally, the susceptibility rates for liver and gall bladder infections was somewhere in between, but it should be noted that the therapeutic effects of antimicrobial drugs can be highly variable when treating different infected organs.

Let’s consider OSWIA > 80% as the initial gold standard. For example, the OSWIA of FOX is around 80% in the appendix, but was < 70% in the other 5 organs examined. Thus, it would only be appropriate for the treatment of specific infections in the appendix. Piperacillin/tazobactam (TZP) is recommended to treat many infections, but OSWIA was only 68.3% in HA pancreas infections, which is inappropriate for empirical treatment. Additionally, we found that apart from liver infections, the weighted susceptibility for each abdominal organ presented as a typical “stair-step” shape, with some of drugs such as ertapenem, amikacin, imipenem and piperacillin/tazobactam being > 80%, and the rest far below this level. The weighted susceptibilities of ertapenem, amikacin, imipenem and piperacillin/tazobactam were highest in all organs, which is in line with another study on Chinese IAIs [13].

ESBL rates of *Enterobacteriaceae* essentially differed between organs (Table 2), which were reflected in the low susceptibility rates to cephalosporins of colon, pancreas and peritoneal fluid isolates (Fig. 2). The high proportion of ESBL-producing strains in the pathogens of the studied organs certainly indicate a high risk for Chinese IAI patients becoming infected with an ESBL producing bacterial strain.

We analyzed the epidemiological data of antimicrobial susceptibilities using an “organ–bacteria–susceptibility” approach, but still a large number of clinical factors could not be included in the analysis, including the drug concentrations at the infection sites and the physical condition of individual patients.

Moreover, drawbacks in our data analysis have been noted. First, a classification based on infected organs reduced the sample size in each group, which decreased the reliability of the statistical analysis, and Gram-negative anaerobes and Gram-positive bacteria were not included. Second, because of the limited strain numbers in each year, we combined data from several years, which might not reflect the actual situation in each year. Yearly OSWIA analysis with sufficient samples should be conducted in large hospitals, as a complement to the traditional model of “bacteria-susceptibility” to support appropriate regimen selection of antibiotics for empirical therapy.

## Conclusions

There are significant variations in the distributions of bacteria in different abdominal organs, with various antimicrobial organ-specific susceptibilities. OSWIA may be used as a complement to the traditional model of “bacteria–susceptibility”, and aid appropriate regimen selection of antibiotics for empirical therapy, particularly for CA IAIs. However, further studies will need to be conducted to validate the correlations between OSWIA, and the cure and survival rates of patients.

## Additional file

Additional file 1: **Table S1.** A total of 61 IAI isolates were collected in < 0.2% of the *Enterobacteriaceae* or < 1.1% of non-fermentative bacterial strains. (DOCX 17 kb)

## Abbreviations

ETP: (ertapenem)
AMK: (amikacin)
IPM: (imipenem)
TZP: (piperacillin/tazobactam)
FOX: (cefoxitin)
CAZ: (ceftazidime)
LVX: (levofloxacin)
FEP: (cefepime)
CIP: (ciprofloxacin)
CRO: (ceftriaxone)
CTX: (cefotaxime)
SAM: (ampicillin/sulbactam)
