# Supplementary material for: Antimicrobial susceptibilities of specific syndromes created with organ-specific weighted incidence antibiograms (OSWIA) in patients with intra-abdominal infections
Source: BMC Infect Dis. 2018 Nov 19;18:584. doi: 10.1186/s12879-018-3494-x (PMC6245934; doi:10.1186/s12879-018-3494-x)
Supplement: Supplementary file 1 — Table S1. A total of 61 IAI isolates were collected in < 0.2% of the Enterobacteriaceae or < 1.1% of non-fermentative bacterial strains. (DOCX 17 kb) [file 12879_2018_3494_MOESM1_ESM.docx]

**Supplementary Table 1.** A total of 61 IAI isolates were collected in < 0.2% of the *Enterobacteriaceae* or < 1.1% of non-fermentative bacterial strains.

*Achromobacter,* non-speciated *1; Acinetobacter anitratus; Acinetobacter calcoaceticus; Acinetobacter junii; Acinetobacter lwoffii; Acinetobacter,* non-speciated*; Aeromonas caviae; Aeromonas sobria; Alcaligenes faecalis; Alcaligenes xylosoxidans; Alcaligenes xylosoxidans ssp; Bacteroides ovatus; Bordetella,* non-speciated*; Burkholderia cepacia; Candida tropicalis; Chryseobacterium meningosepticum; Chryseomonas luteola; Citrobacter amalonaticus; Citrobacter braakii; Citrobacter diversus; Citrobacter koseri; Citrobacter youngae; Citrobacter,* non-speciated*; Comamonas testosterone; Cronobacter sakazakii; Elizabethkingia meningoseptica; Enterobacter agglomerans; Enterobacter amnigenus I; Enterobacter asburiae; Enterobacter gergoviae; Enterobacter hormaechi; Enterobacter intermedium; Enterobacter,* non-speciated*; Flavobacterium meningosepticum; Flavobacterium,* non-speciated*; Hafnia alvei; Klebsiella aerogenes; Klebsiella ornithinolytica; Klebsiella ozaenae; Klebsiella planticola;* Non lactose fermenting gram negative rods; *Ochrobactrum,* non-speciated*; Proteus penneri; Proteus vulgaris*

*Providencia rettgeri; Providencia stuartii; Pseudomonas alcaligenes; Pseudomonas cepacia; Pseudomonas fluorescens; Pseudomonas maltophilia; Pseudomonas mendocina; Pseudomonas putida; Pseudomonas stutzeri; Pseudomonas,* non-speciated*; Salmonella enteritidis; Salmonella typhi; Salmonella,* non-speciated*; Serratia fonticola; Serratia liquefaciens; Serratia odorifera; Shigella flexneri*
